# IL-6 Trans-Signaling in the Brain Influences the Metabolic Phenotype of the 3xTg-AD Mouse Model of Alzheimer’s Disease

**DOI:** 10.3390/cells9071605

**Published:** 2020-07-02

**Authors:** Anna Escrig, Amalia Molinero, Brenda Méndez, Mercedes Giralt, Gemma Comes, Paula Sanchis, Olaya Fernández-Gayol, Lydia Giménez-Llort, Christoph Becker-Pauly, Stefan Rose-John, Juan Hidalgo

**Affiliations:** 1 Institute of Neurosciences and Department of Cellular Biology, Physiology and Immunology, Faculty of Biosciences, Universitat Autònoma de Barcelona, 08193 Bellaterra, Spain; annesmon@gmail.com (A.E.); Amalia.Molinero@uab.cat (A.M.); bren.mendezm@gmail.com (B.M.); merce.giralt@uab.cat (M.G.); Gemma.comes@uab.cat (G.C.); paula.sanchis@uab.cat (P.S.); of2194@cumc.columbia.edu (O.F.-G.); 2 Institute of Neurosciences and Department of Psychiatry and Forensic Medicine, Faculty of Medicine, Universitat Autònoma de Barcelona, 08193 Bellaterra, Spain; lidia.gimenez@uab.cat; 3 Department of Biochemistry, Medical Faculty, Christian-Albrechts-Universität zu Kiel, 24098 Kiel, Germany; cbeckerpauly@biochem.uni-kiel.de (C.B.-P.); rosejohn@biochem.uni-kiel.de (S.R.-J.)

**Keywords:** Alzheimer’s disease, IL-6, IL-6 trans-signaling, body weight, high-fat diet, insulin, leptin

## Abstract

Alzheimer’s disease (AD) is a neurodegenerative disorder that causes the most prevalent dementia in the elderly people. Obesity and insulin resistance, which may cause major health problems *per se*, are risk factors for AD, and cytokines such as interleukin-6 (IL-6) have a role in these conditions. IL-6 can signal either through a membrane receptor or by trans-signaling, which can be inhibited by the soluble form of the co-receptor gp130 (sgp130). We have addressed the possibility that blocking IL-6 trans-signaling in the brain could have an effect in the triple transgenic 3xTg-AD mouse model of AD and/or in obesity progression, by crossing 3xTg-AD mice with GFAP-sgp130Fc mice. To serve as control groups, GFAP-sgp130Fc mice were also crossed with C57BL/6JOlaHsd mice. Seventeen-month-old mice were fed a control diet (18% kcal from fat) and a high-fat diet (HFD; 58.4% kcal from fat). In our experimental conditions, the 3xTg-AD model showed a mild amyloid phenotype, which nevertheless altered the control of body weight and related endocrine and metabolic factors, suggestive of a hypermetabolic state. The inhibition of IL-6 trans-signaling modulated some of these traits in both 3xTg-AD and control mice, particularly during HFD, and in a sex-dependent manner. These experiments provide evidence of IL-6 trans-signaling playing a role in the CNS of a mouse model of AD.

## 1. Introduction

Alzheimer’s disease (AD) is a neurodegenerative disorder that causes the most prevalent form of dementia in elderly people. Clinically, it courses with impaired cognitive functions and memory loss caused mainly by the accumulation of β-amyloid peptides (Aβ) forming extracellular plaques and the intracellular aggregation of hyperphosphorylated Tau protein forming neurofibrillary tangles [1]. Both features cause a prominent neuroinflammation in the central nervous system (CNS) with marked microglia and astroglia activation and the release of several pro-inflammatory cytokines [2].

Multiple risk factors have been associated with AD; as aging is the most important, the accumulation of mtDNA mutations and mitochondrial dysfunction caused by ROS could be an early event in the disease [3]. Additionally, AD is also linked to some metabolic alterations. Being overweight in middle age (40–45 years) increases the risk of dementia, at older ages (65–75 years) the relation is U shaped, whereas at ≥76 years a higher BMI is related to decreased risk [4]. Accordingly, weight loss is commonly associated with AD patients [5]. Chronic systemic inflammation is also a landmark of obesity with secretion of systemic-acting pro-inflammatory cytokines from the adipose tissue, which can even gain access to the brain through the blood brain barrier [6]. Some studies using diet-induced obesity rodent models demonstrate impairment in memory and learning [7,8], reduced synaptic plasticity [9] and eventually compromised neurogenesis [10]. These effects may be caused by the infiltration of cytokines from the periphery and by local neuroinflammatory processes and subsequent alteration of insulin pathways in the brain [11,12]. Additionally, an altered insulin signaling in the brain contributes to Aβ aggregation and increases hyperphosphorylated tau protein levels [13]. 

Interleukin-6 (IL-6) is one of the potential cytokines involved in the inflammatory processes previously explained [14,15]. It has a complex signaling pathway mediated via three distinct modes. In the classic signaling, IL-6 binds to a specific membrane receptor (mIL-6R), which activates dimerization of gp130 and subsequent downstream JAK/STAT signaling. In the trans-signaling, IL-6 binds to a soluble form of mIL-6R (sIL-6R) to activate gp130. Due to the ubiquitous expression of gp130, most cells in the body can be stimulated by this process. IL-6 trans-signaling is specifically inhibited by the soluble form of gp130, sgp130 [16]. The third pathway called cluster signaling has been described as an exclusive mechanism for the communication between dendritic and T cells [17]. 

The implication of IL-6 in the control of body weight and metabolism has been reported previously. Wallenius and colleagues demonstrated that IL-6 KO mice develop mature-onset obesity and decreased glucose tolerance [18]. Accordingly, mice with overexpression of this cytokine in astrocytes (GFAP-IL6 mice) are resistant to high-fat diet (HFD)-induced obesity [19]. The development of conditional KO mice for different brain cells also revealed alterations in the control of body weight [20,21]. Interestingly, IL-6 trans-signaling has also been implicated in obesity, as sIL-6R is a critical chemotactic signal for adipose tissue macrophages recruitment [22]. 

Classic signaling is crucial for anti-inflammatory IL-6 functions; in contrast, IL-6 trans-signaling is more related to its pro-inflammatory actions, being involved in some neurodegenerative diseases such as AD and Parkinson’s disease, inflammatory colon cancer, arthritis and inflammatory bowel disease [23]. It has been demonstrated that blocking IL-6 trans-signaling with the sgp130Fc protein decreases inflammation-related damage in peripheral tissues [22] and in the CNS (by using the so-called GFAP-sgp130Fc mice, which express sgp130Fc in astrocytes [24]). Additionally, it has been discovered that the common IL-6R variant p.D358A (rs2228145), which increases its proteolysis by ADAM10 and ADAM17 and as a result increases the amount of sIL-6R, may advance the age of onset of AD in APOE ε4 carriers [25].

We hypothesized that blocking IL-6 trans-signaling with sgp130Fc in the brain would impact AD and/or obesity progression. We recently demonstrated the former in two different mouse models of AD, namely the 3xTg-AD mice and the Tg2576 mice, by crossing them with GFAP-sgp130Fc mice in order to obtain 3xTg-AD mice with the IL-6 trans-signaling pathway blocked in the CNS [26]. We herewith present results with 3xTg-AD mice, with and without transgenic sgp130Fc expression by astrocytes, and challenged with a high-fat diet to elicit obesity, which suggest that IL-6 trans-signaling modulates some of the hypermetabolic traits presented by the 3xTg-AD mice in a sex-dependent manner. 

## 2. Materials and Methods

### 2.1. Generation of Double Transgenic 3xTg-AD/GFAP-sgp130Fc Mice

The parental strains used in this study, GFAP-sgp130Fc and 3xTg-AD mice, were generated and characterized previously [24,27,28]. To obtain the 4 genotypes of interest the following crossing strategy was designed, as in [26]. Homozygous 3xTg-AD (+/+) animals were mated with heterozygous GFAP-sgp130Fc (+/−) to obtain the double transgenic 3xTg-AD/GFAP-sgp130Fc (+/−) (+/−) and 3xTg-AD (+/−) (−/−) mice. In addition, GFAP-sgp130Fc (+/−) mice were also crossed with WT mice (C57BL/6JOlaHsd) to obtain the control groups GFAP-sgp130Fc (+/−) and WT (−/−). Genotype was determined by PCR analysis of tail DNA. (*sgp130*Fw: 5’-GAGGTGACATGTGTGGTGG TGGATGTTC-3’; *sgp130*Rv: 5’-GAGAACACGTTGCCCTGCTGCCATCTAG-3’/*App*Fw: 5’-GCTTGC ACCAGTTCTGGATGG-3’; *App*Rv: 5’-GAGGTATTCAGTCATGTGCT-3’/*Tau*Fw: 5’-GAGGTATT CAGTCATGTGCT-3’; *Tau*Rv: 5’-TTCAAAGTTCACCTGATAGT-3’).

All mice used in this study were fed ad libitum (unless otherwise stated) and housed in a 12 h dark-light cycle under specific pathogen-free conditions and constant temperature (22 ± 2 °C). All experimental procedures were approved by the Ethics Committee in Human and Animal experimentation (CEEAH 3541 and 3971, valid up to 06/2019 and 05/2023, respectively) from the Autonomous University of Barcelona and were in accordance with Spanish legislation and the EU directive (2010/63/UE) on ‘Protection of Animals Used for Experimental and Other Scientific Purposes’. 

Animals were sacrificed by decapitation to collect the brain. The right hemisphere was dissected into cortex, hippocampus and hypothalamus and flash-frozen in liquid nitrogen for gene expression and ELISA. Blood was collected from the trunk and centrifuged (9520 g for 10 minutes) at 4 °C to obtain the serum. All samples were stored at −80 °C until processed. Subsets of animals were sacrificed at different ages to check disease progression. 

### 2.2. Diets

The HFD (TD 03584, 58.4% kcal from fat), and the control diet (2018, 18% kcal from fat) were obtained from Harlan Iberica (Sant Feliu de Codines, Spain). The experiment had two phases and began when the mice were 17-month-old: they were first fed the control diet (*n* = 6–11); and then the HFD. Because of aging-related mortality, seven new 3xTg-AD male mice were added in the second phase, which showed similar responses to the other mice. Body weight and food intake were monitored regularly. In most cases mice were not isolated but kept group-housed after weaning (2–3 per cage) according to their genotype and sex. During the control diet phase, animals were subjected sequentially to an insulin tolerance test (ITT), a fasting and refeeding test (F&R), and an oral glucose tolerance test (OGTT), on weeks 3, 4 and 5, respectively. During the HFD phase, mice were subjected to ITT and OGTT only, in the weeks 6 and 8, respectively. Hypothalamus, liver, visceral (gonadal) and subcutaneous (inguinal) fat depots and brown adipose tissue were removed and weighed upon euthanasia of the animals and frozen in liquid nitrogen for further analysis. Body temperature was measured at the beginning of the control diet phase.

### 2.3. Body Temperature 

Body temperature measurement was carried out using a lubricated rectal probe (Cibertec, Madrid, Spain). The measurements were taken throughout the day, at 8, 11, 16 and 19 h.

### 2.4. Fasting and Refeeding (F&R)

The animals were fasted overnight for 14 h and posteriorly refed. Body weight and food intake were measured regularly during the first 48 h post-fasting. Using these parameters body weight change post-fasting was calculated as well as the food intake.

### 2.5. Insulin Tolerance Test (ITT) and Oral Glucose Tolerance Test (OGTT)

The metabolic status of the mice was evaluated by an insulin tolerance test (ITT) and an oral glucose tolerance test (OGTT). For ITT, mice were injected intraperitoneally (i.p.) with 1 U/kg of insulin after a 4 h fast. For OGTT, mice were fasted for 15 h before being administered an oral glucose dose of 2 g/kg of body weight. Blood glucose was measured in tail samples at time points 0, 15, 60 and 120 min for ITT and at 0, 30, 60 and 120 min for OGTT with an ACCU CHECK glucometer (Roche).

### 2.6. Enzyme-Linked ImmunoSorbent Assays (ELISAs) and Colorimetric Tests

ELISA kits were used to measure soluble Aβ_40_, Aβ_42_ and sgp130Fc levels in brain samples (Amyloid beta 40 and 42 Human ELISA Kits, ThermoFisher (Vienna, Austria); Human sgp130 DuoSet ELISA, R&D Systems (Abingdon, UK) and leptin, insulin and FGF-21 in serum (rat/mouse ELISA leptin/insulin kits, Millipore (Billerica, MA, USA), ; Mouse/Rat FGF-21, R&D Systems (Minneapolis, MN, USA). In the case of Aβ measurements, brain samples were homogenized by sonication in a lysis buffer (50 nM TrisHCl, 150 nM NaCl, 2 mM EDTA, 0.1% Igepal CA 630 (Fluka, St. Louis, MO, USA, 3% SDS and 2% sodium deoxycolate) supplemented with a protease inhibitor cocktail and phenylmethylsulfonyl fluoride (Sigma-Aldrich, St. Louis, MO, USA). For sgp130Fc, brain samples were homogenized using a tissue homogenizer (Precellys, Bertin Instruments, Montigny-le-Bretonneux, France) in RIPA buffer supplemented with phosphatase and protease inhibitors. To measure cholesterol, triglycerides and glucose in serum specific colorimetric test kits were used (Cholesterol MonlabTest^®^/Triglycerides MonlabTests^®^ (Selva de Mar, Spain); and Glucose-TR Spinreact (Sant Esteve de Bas, Spain).

### 2.7. RT-qPCR

For the two-step RT-qPCR, total RNA was isolated using Maxwell simply RNA Tissue Kit (Promega, Madison, WI, USA) from hypothalamus and liver. Then, it was reverse transcribed into cDNA in a reaction using iScript cDNA synthesis kit and iTaq Universal SYBR Green Supermix (Bio-Rad Laboratories, Inc., Hercules, CA, USA). RT-qPCR for selected genes important for this study (*Il6* and *Agrp* in hypothalamus and *Fgf21* in liver) was performed in 384-well plates and run in CFX384 real-time PCR detection system (Bio-Rad Laboratories, Inc., Hercules, CA, USA). *Gapdh* was used as the reference gene [29,30]. (*Il6*Fw: 5’-GCTTA ATTACACATGTTCTCTGGGAAA-3’; *Il6*Rv: 5’-CAAGTGCATCATCGTTGTTCATAC-3’/*Agrp*Fw: 5’-AGAGTTCCCAGGTCTAAGTCTG-3’; *Agrp*Rv: 5’-GCGGTTCTGTGGATCTAGC-3’/*Fgf21Fw:* 5’-TTCTTTGCCAACAGCCAGAT-3’; *Fgf21Rv*: 5’-GTCCTCCAGCAGCAGTTCTC-3’/*Gapdh*Fw: 5’-GGCAAATTCAACGGCACA-3’; *Gapdh*Rv: 5’-CGGAGATGATGACCCTTT-3’).

### 2.8. Statistics

Statistical calculations were done using the Statistical Package for Social Sciences version 19 (SPSS, Chicago, IL, USA) and RStudio (v 1.2.5033, R version 4.0.1, Boston, MA, USA). Males and females were analyzed separately. Most data were analyzed using the generalized linear model (GLZ) with 3xTg-AD (positive *vs.* negative) and sgp130Fc (positive *vs.* negative) as main factors; when there is a significant interaction between the main factors in the GLZ it is stated accordingly. Generalized estimated equations (GEE) were used for repeated measures (i.e., body weight, temperature, ITT and OGTT) including also both genotypes as main factors and time as a within-subject factor. In case of comparisons between two groups, an unpaired t-Student was used. Effect size was calculated using Glass’ delta (with R’s *effectsize* package, with small sample correction) for unpaired t-tests. For GLZ data, we indicate *R*^2^ (Equation. (1), [31]) as a measure of the variance explained by the model. Representative % of changes between 3xTg-AD and WT groups (unless otherwise stated) will be stated in some cases. Statistical significance was defined as *p* ≤ 0.05.

Equation (1). *R*^2^, or coefficient of determination. The numerator, $\sigma_{\varepsilon }^{2}$, indicates the variance of the residuals, and the denominator is the variance of the dependent variable:

(1) $$R^{2}=1-\frac{\sigma_{\varepsilon }^{2}}{\sum_{i=1}^{n}{\left(y_{i}-\bar{y}\right)}^{2}}$$

## 3. Results

### 3.1. sgp130Fc Influenced Body Weight and Food Intake in a Sex-Dependent Manner in Response to a High-Fat Diet 

**Figure 1:**
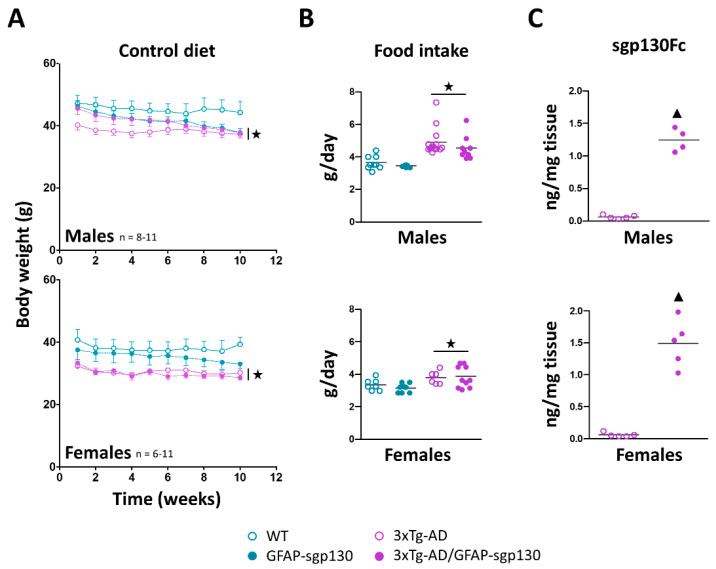
Body weight and food intake alterations in control diet. (**A**) During the control diet phase, 3xTg-AD mice presented lower body weight. The inhibition of the IL-6 trans-signaling did not have a significant effect. (**B**) 3xTg-AD mice presented increased food intake in both sexes, with no effect of the sgp130Fc protein. (**C**) Sgp130Fc quantification by ELISA in brain cortex of AD mice. Results are MEAN ± SEM. ★ *p* ≤ 0.05 vs. 3xTg-AD negative; ▲ *p* ≤ 0.05 vs. sgp130Fc negative.

Having in mind that during the control diet phase the mice were subjected to an ITT, a F&R and an OGTT, the results indicate that 3xTg-AD animals had lower body weight (decreasing for instance a ~13.5% and ~21.3% in 3xTg-AD vs WT in male and female mice, respectively). This decrease was significant when comparing the four groups in both sexes (males *p* = 0.039; females *p* < 0.001), whereas the inhibition of IL-6 trans-signaling did not have a clear effect (Figure 1A). Despite their lower body weight, absolute (g/day) food intake was significantly increased in 3xTg-AD mice (increasing ~25.6% and 12.8% in 3xTg-AD mice vs WT mice in male and female mice, respectively) (males *p* < 0.001 *R*^2^ = 0.54; females *p* = 0.002 *R*^2^ = 0.33) (Figure 1B). No significant effect of the sgp130Fc protein was observed.

**Figure 2:**
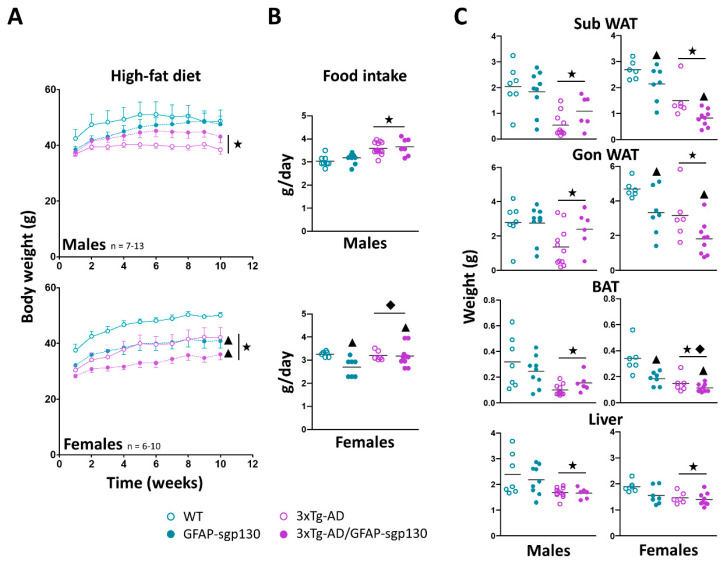
Body weight and food intake alterations in high-fat diet. (**A**) HFD caused obesity, and again 3xTg-AD mice presented lower body weight. The inhibition of IL-6 trans-signaling did not have a significant effect in male mice, whereas in females it decreased HFD-induced obesity of both 3xTg-AD and control mice. (**B**) 3xTg-AD male mice had also higher food intake in this phase. This effect was not observed in females. The inhibition of IL-6 trans-signaling had no effects on males, in females, it decreased food intake, more markedly in the WT than in 3xTg-AD animals. (**C**) The absolute weight from the adipose tissue depots and liver was diminished in 3xTg-AD mice. The blocking of IL-6 trans-signaling further diminished the absolute tissue weights in females. Results are MEAN ± SEM. ★ *p* ≤ 0.05 vs. 3xTg-AD negative; ▲ *p* ≤ 0.05 vs. sgp130Fc negative; ◆ Indicates a significant interaction between both factors.

Despite their advanced age, in general mice gained weight during the HFD phase, and as seen with the control diet, with HFD the 3xTg-AD mice again had lower body weight (decreasing a ~18.5% and ~16.0% in 3xTg-AD vs WT in male and female mice, respectively). This decrease was significant when comparing the four groups (males *p* = 0.009; females *p* < 0.001) (Figure 2A). Additionally, 3xTg-AD male mice presented higher food intake (~18.2% in 3xTg-AD vs WT) (*p* < 0.001 *R*^2^ = 0.50) (Figure 2B). The inhibition of IL-6 trans-signaling did not have a significant effect on the body weight in male mice, although, it is likely that this rather indicates that the 3xTg-AD/GFAP-sgp130Fc male mice have recovered better from the different experimental procedures they were subjected to. Thus, at the end of the HFD phase, a similar body weight difference (compared to 3xTg-AD mice) to that observed at the beginning of the control phase is now apparent. In contrast, in females the inhibition of IL-6 trans-signaling decreased HFD-induced obesity of both 3xTg-AD and control mice (*p* < 0.001). Results for food intake were similar to those of the control diet phase, but significant interaction emerged since it was increased in 3xTg-AD/GFAP-sgp130Fc (~17.8% vs GFAP-sgp130Fc) but not in 3xTg-AD (vs WT) females (*p* = 0.049 *R*^2^ = 0.50).

The weights of liver and fat tissues (Figure 2C) were in concordance with those of body weight. Thus, all of them were decreased (from ~29 to 74% in 3xTg-AD vs WT) significantly in 3xTg-AD male (WATSc *p* < 0.001 *R*^2^ = 0.50; WATGon *p* = 0.032 *R*^2^ = 0.27; BAT *p* = 0.001 *R*^2^ = 0.38; Liver *p* = 0.002 *R*^2^ = 0.31) and female (from ~23 to 56% in 3xTg-AD vs. WT) mice (WATSc *p* < 0.001 *R*^2^ = 0.69; WATGon *p* = 0.002 *R*^2^ = 0.50; BAT *p* < 0.001 *R*^2^ = 0.64; Liver *p* = 0.008 *R*^2^ = 0.37), and the inhibition of IL-6 trans-signaling further decreased them in all females (WATSc *p* < 0.005 ; WATGon *p* = 0.005; BAT *p* = 0.001). 

The presence of sgp130Fc was measured in brain cortex from 3xTg-AD and 3xTg-AD/GFAP-sgp130Fc mice at 10 months of age by ELISA (Figure 1C). As expected, a clear increase in sgp130Fc immunostaining was observed in 3xTg-AD/GFAP-sgp130Fc mice (males *p* = 0.001 Glass’ delta = 33.42; females *p* = 0.001 Glass’ delta = 34.42), with no differences between males and females.

### 3.2. 3xTg-AD Mice Recovered the Body Weight Quicker and Had a Higher Food Intake after an Overnight Fasting. The Inhibition of IL-6 Trans-Signaling Attenuated These Effects Especially in 3xTg-AD Males

**Figure 3:**
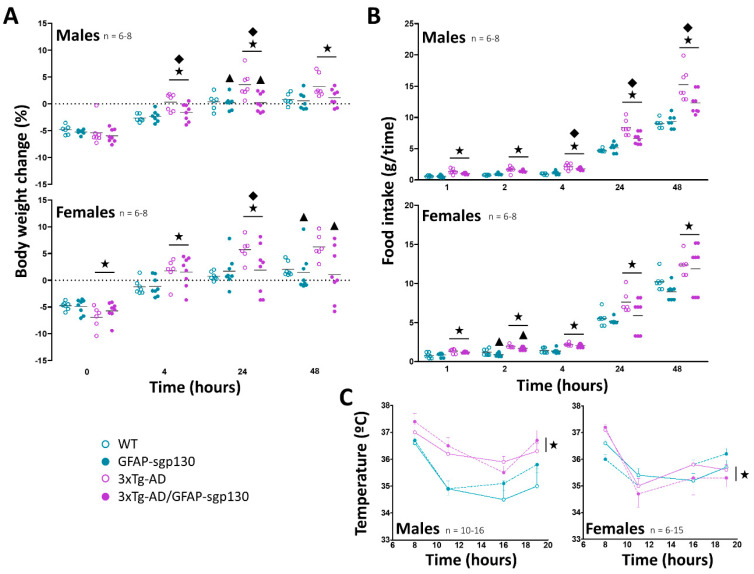
Fasting and refeeding analysis and basal temperature in mice fed with control diet. (**A**) After fasting, 3xTg-AD mice lost more weight than controls (significant in females), without effects of the IL-6 trans-signaling inhibition. Once the animals were re-fed, 3xTg-AD animals gained weight quicker than controls, a response blunted by the blocking of IL-6 trans-signaling. (**B**) A post-fasting hyperphagia was observed in the 3xTg-AD mice, a response again blunted in some cases by the blocking of IL-6 trans-signaling. (**C**) Higher body temperatures were observed in the earlier hours of the day, when 3xTg-AD mice showed an increase compared with controls. This effect was maintained throughout the day in males. No effect of the inhibition of the IL-6 trans-signaling was observed. Results are MEAN ± SEM. ★ *p* ≤ 0.05 vs. 3xTg-AD negative; ▲ *p* ≤ 0.05 vs. sgp130Fc negative; ◆ Indicates a significant interaction between both factors.

As expected, all mice lost weight after an overnight fasting (Figure 3A). In females, this loss was higher in 3xTg-AD mice regardless of sgp130Fc expression (*p* = 0.015 *R*^2^ = 0.25). No differences were observed in males. Once the animals were re-fed their body weight increased; the 3xTg-AD mice recovered faster in both sexes (males t4 *p* = 0.001 *R*^2^ = 0.45, t24 *p* = 0.029 *R*^2^ = 0.42, t48 *p* = 0.026 *R*^2^ = 0.32; females t4 *p* = 0.002 *R*^2^ = 0.33, t24 *p* = 0.032 *R*^2^ = 0.28), a response blunted by the blocking of IL-6 trans-signaling, especially in males. Absolute food intake measurements (Figure 3B) showed a compensatory hyperphagia in 3xTg-AD mice after fasting, evident in both sexes (males *p* < 0.001 *R*^2^ = 0.66; females *p* < 0.001 *R*^2^ = 0.49), a response again blunted by sgp130Fc in male mice, and to a limited extent in female mice. 

Regarding body temperature (Figure 3C), it oscillated according to the circadian rhythm between 1 and 3 °C throughout the day. The higher temperatures were observed in the earlier hours of the day when it was more evident that 3xTg-AD mice showed higher body temperature compared with control mice in both sexes (males *p* < 0.001; females *p* = 0.034). However, only 3xTg-AD males kept the same trend throughout the day. Blocking IL-6 trans-signaling did not cause any effect.

### 3.3. 3xTg-AD Mice Showed Lower Hormonal and Cholesterol Levels in Serum, Although There Were No Differences in Glycaemia 

**Figure 4:**
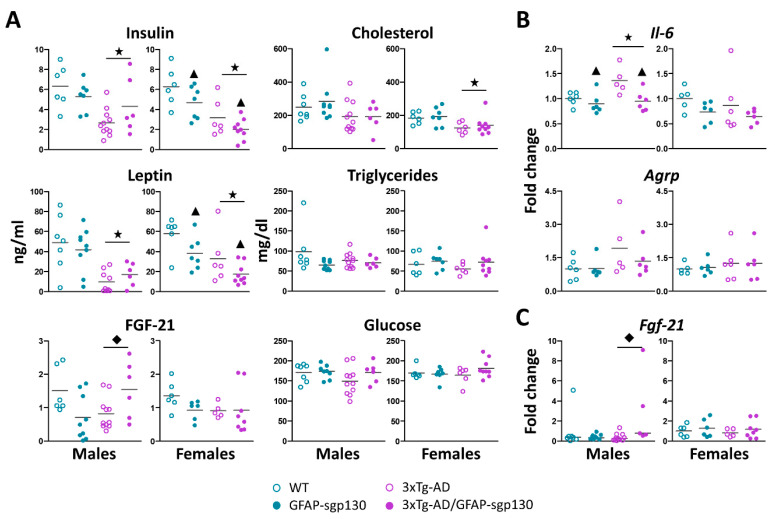
Hormones and metabolites in serum. Hypothalamic and liver gene expression after HFD. (**A**) Generally, 3xTg-AD mice showed lower serum levels of insulin, leptin and cholesterol. Blocking of IL-6 trans-signaling decreased insulin and leptin levels in 3xTg-AD and control females and increased Fgf-21 levels in 3xTg-AD males. (**B**) *Il6* expression in the hypothalamus was increased in 3xTg-AD males; and this effect was rescued by the presence of sgp130Fc. No differences were seen in the orexigenic *Agrp*. (**C**) *Fgf21* expression in the liver was increased by the inhibition of IL-6 trans-signaling in 3xTg-AD males; no effects were observed in females. Results are MEAN ± SEM. ★ *p* ≤ 0.05 vs. 3xTg-AD negative; ▲ *p* ≤ 0.05 vs. sgp130Fc negative; ◆ Indicates a significant interaction between both factors.

Figure 4A shows the hormones and blood metabolites after the HFD in all genotypes and both sexes. 3xTg-AD females presented lower (~32.3% in 3xTg-AD vs WT) cholesterol (*p* = 0.006 *R*^2^ = 0.28), whereas triglycerides and glucose were normal; no effect of IL-6 trans-signaling was observed in these parameters. In addition, there was a general trend for 3xTg-AD animals having decreased levels of insulin, leptin and FGF-21. For instance, insulin levels were decreased a ~57.8% (male) and ~42.2% (female) in 3xTg-AD vs WT mice. When analyzed the four groups together, significant differences were clearly observed (males *p* = 0.003 *R*^2^ = 0.39; females *p* < 0.001 *R*^2^ = 0.55). Significant differences were also observed for leptin (decreasing a ~79.9% (male) and ~43.2% (female) in 3xTg-AD vs WT) (males *p* < 0.001 *R*^2^ = 0.46; females *p* = 0.002 *R*^2^ = 0.46) and FGF-21 (from ~33.1 to 45.3% in 3xTg-AD vs WT). In males, blocking of IL-6 trans-signaling tended to reverse these 3xTg-AD-elicited changes (FGF-21 *p* = 0.004 *R*^2^ = 0.27); in females, in contrast, it tended to further decrease them regardless of the 3xTg-AD genotype (insulin *p* = 0.031; leptin *p* = 0.014). 

For analyses of gene expression, a RT-qPCR was carried out in the hypothalamus (Figure 4B) and liver (Figure 4C). In the hypothalamus, no differences were obtained between the different genotypes in *Agrp,* whereas *Il6* mRNA levels were increased in 3xTg-AD males (a ~36% in 3xTg-AD vs WT) (*p* = 0.026 *R*^2^ = 0.47), an effect reversed by the inhibition of the IL-6 trans-signaling (*p* = 0.007). In liver, the inhibition of IL-6 trans-signaling elevated significantly *Fgf21* hepatocyte gene expression in the AD positives males (*p* = 0.033 *R*^2^ = 0.1); no differences were observed in females.

### 3.4. 3xTg-AD Mice Had Hypersensitivity to Insulin and Increased Tolerance to Glucose Measured by ITT and OGTT, Respectively 

**Figure 5:**
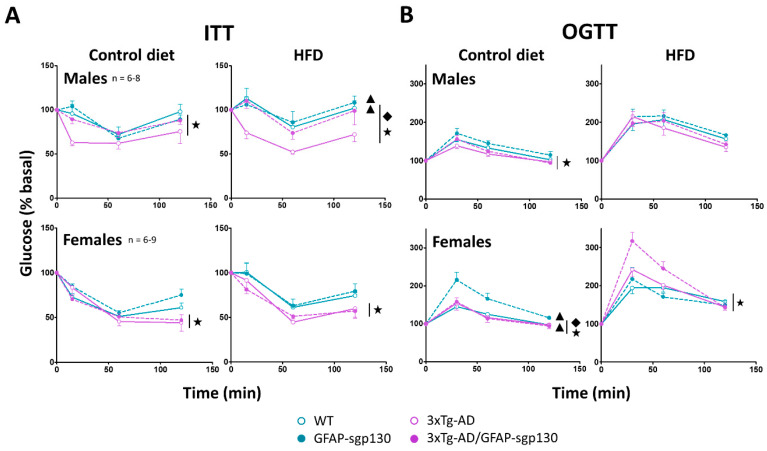
Insulin tolerance test (ITT) and oral glucose tolerance test (OGTT) at both experimental phases. (**A**) 3xTg-AD mice showed hypersensitivity to insulin in both control and HFD phases, which was reverted by the inhibition of IL-6 trans-signaling in males during the HFD phase. (**B**) In the control phase, the 3xTg-AD mice tended to have better glucose tolerance, whereas in the HFD phase results were not clear-cut; this was also the case regarding the effect of sgp130Fc. Results are MEAN ± SEM. ★ *p* ≤ 0.05 vs. 3xTg-AD negative; ▲ *p* ≤ 0.05 vs. sgp130Fc negative; ◆ Indicates a significant interaction between both factors.

Insulin sensitivity and glucose tolerance during both the control and HFD phases are shown in Figure 5A,B, respectively. Insulin sensitivity was increased in 3xTg-AD mice in both sexes (males *p* = 0.039; females *p* = 0.034) and blocking of IL-6 trans-signaling did not reach statistical significance. During HFD sensitivity was still higher in 3xTg-AD mice (males *p* = 0.005; females *p* = 0.008), but in this case the effect of sgp130Fc significantly reversed it in male mice (*p* = 0.047).

In accordance with their increased insulin sensitivity, glucose tolerance was better in 3xTg-AD mice (males *p* = 0.010; females *p* = 0.028), during the control diet (Figure 5B). During HFD, however, that was not significant in male mice, and somewhat surprisingly, in females the opposite emerged (*p* = 0.005). In females with control diet, the blocking of IL-6 trans-signaling decreased this tolerance (*p* = 0.032) mostly in control animals as indicated by the significant interaction between genotypes (*p* = 0.027), whereas during HFD no significant effects were observed in 3xTg-AD animals.

### 3.5. Mild Phenotype of 3xTg-AD Mice

**Figure 6:**
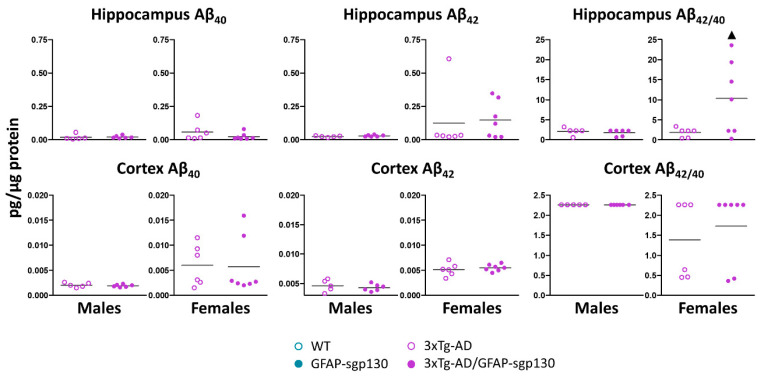
Presence of Aβ in brain of 3xTg-AD mice. Low levels of Aβ were detected in cortex and hippocampus from 3xTg-AD mice fed with HFD. The inhibition of the IL-6 trans-signaling significantly increased the ratio Aβ_42_/Aβ_40_ in the hippocampus. Results are MEAN ± SEM. ▲ *p* ≤ 0.05 vs. sgp130Fc negative.

Soluble Aβ_40_ and Aβ_42_ levels in cortex and hippocampus from males and females were measured following HFD by ELISA (Figure 6). Both were very low in both 3xTg-AD and 3xTg-AD/GFAP-sgp130Fc mice, and several mice showed undetectable levels; conservatively, we assigned a value equivalent to the lowest one measured in the ELISA for these animals. In general, female mice showed higher Aβ_40_ and Aβ_42_ levels than males, and the blocking of the IL-6 trans-signaling did not have significant effects. Nevertheless, it significantly increased (a ~463% in 3xTg-AD/GFAP-sgp130Fc vs 3xTg-AD) the ratio Aβ_42_/Aβ_40_ in the hippocampus (*p* = 0.05 Glass’ delta = 6.33).

## 4. Discussion

AD and obesity are major public health problems. They are two of the most prevalent diseases in our society and understanding their pathological mechanisms and the relationship between them will help us to develop successful therapies. We have analyzed the putative interaction between AD transgenes and HFD-induced obesity in a widely used AD mouse model, with and without the blocking of IL-6 trans-signaling in the brain.

We previously showed that 3xTg-AD male and female mice fed a normal diet have a diminished body weight [26]. This is also the case with other AD mouse models, such as the Tg2576 [26,32,33,34,35]. This is similar to the loss of weight present in AD patients [5]. Therefore, it was not unexpected to find that 3xTg-AD mice were again weighing less than controls, regardless of sex. Importantly, this was also the case when fed a HFD. This lower body weight was accompanied by (i) a diminished mass of the white and brown adipose tissues and liver; (ii) decreased circulating levels of cholesterol and of insulin, leptin and FGF-21 serum levels, hormones that are expected to be regulated according to body weight and fat and/or liver mass; and (iii) with the increased insulin sensitivity in the ITT and glucose tolerance in the OGTT [36,37,38]. Contrary to expectations, 3xTg-AD mice had a higher food intake during both phases. This increased food intake, despite lower body weight, could be the consequence of the endocrine changes observed, since leptin and insulin inhibit food intake by acting in hypothalamic nuclei. Previous studies demonstrated that the hyperphagia in the 3xTg-AD model could be attributed to a defective gut-brain signaling, with a reduction in the sensitivity to the anorexic actions of certain satiety factors such as cholecystokinin (CKK) [39]. We challenged the mice in the control diet phase with an overnight fasting, and observed that the 3xTg-AD mice lost more weight, but upon refeeding they regained weight faster than control mice, likely because they had an increased drive for food intake. These results strongly suggest that the 3xTg-AD mice present a hypermetabolic state, which would reconcile their lower body weight with the higher food intake. Indeed, their body temperature throughout the day was also significantly increased. Interestingly, all these effects were more evident in 3xTg-AD male mice than in females. These results are in agreement with previous findings where 3xTg-AD animals presented a hypermetabolic state, the reduction in body weight being accompanied by a significant rise in metabolic rate indicated by higher oxygen consumption and carbon dioxide production [40,41,42]. 

Previous studies demonstrated the implication of IL-6 in body weight regulation, likely by acting on critical hypothalamic and/or other central systems. Thus, IL6-KO mice develop mature-onset obesity [18], whereas transgenic mice overexpressing IL-6 in astrocytes (GFAP-IL6 mice) are resistant to HFD-induced obesity [19]. A recent study demonstrated that IL-6 trans-signaling is enhanced in the CNS of obese mice, that the central administration of IL-6 suppresses feeding, and that the co-injection of sgp130Fc and IL-6 abrogated the food-intake-suppressing effect of IL-6 in both lean and HFD-fed obese animals [43]. In our physiologically relevant experiments (i.e., no injection of IL-6 centrally), we did notice that during the control diet phase, the transgenic expression of sgp130Fc by astrocytes caused a modest decrease of absolute food intake of control male and female mice. Following an overnight fasting, body weight regain was also diminished by the blocking of IL-6 trans-signaling in the brain in 3xTg-AD mice of both sexes, at least in part because of a decreased food intake upon refeeding. Interestingly, the effect of central sgp130Fc is more prominent in 3xTg-AD mice than in controls when fed a control diet. We next studied the effect of a HFD, where significant differences in food intake caused by sgp130Fc were not noticed in female mice. Unfortunately, a F&R experiment could not be carried out in this phase. However, HFD-induced obesity was clearly decreased in female mice, regardless of 3xTg-AD transgenes. This again suggests that blocking IL-6 trans-signaling in the brain abrogates central effects of IL-6 as proposed by Timper et al. [43]. Since this effect is the opposite of what is observed in IL6-KO mice [18], it is presumably revealing a major difference between IL-6 trans-signaling and IL-6 membrane receptor-mediated normal signaling in the brain. Kraakman and colleagues described that mice with peripheral IL-6 trans-signaling inhibition had an increase in body weight, fat mass and leptin levels in serum, although the presence of sgp130Fc prevents (HFD)-induced adipose tissue macrophages accumulation [22]. This underlines the fundamental difference of blocking IL-6 trans-signaling in the CNS compared to the periphery. A role of IL-6 in the hypothalamic systems controlling food intake and energy expenditure has been suggested extensively [21,44,45,46]. In our experiment, however, the expression of *Agrp* was not significantly altered in the situations studied. The changes observed in insulin, leptin and FGF-21 levels mostly suggest that they are secondary to changes in body weight, fat depots and liver. 

As expected [26], 3xTg-AD animals presented a mild phenotype. Soluble Aβ_40_ and Aβ_42_ levels were very low (~an order of magnitude below those of Tg2576 mice [32,33]) at an advanced age (~ 20 months), in both cortex and hippocampus. Despite the technical difficulties (some mice were below the limit of sensibility of the ELISA), by comparing the present results with those of our earlier study with a control diet [26], we noticed that Aβ levels are further decreased by HFD. The reason for this is unclear, since previous studies do not support such an inhibitory effect of HFD in 3xTg-AD [41,47] and Tg2576 [48]. This mild phenotype may have to do with (i) the fact that we used hemizygous mice because of the experimental design (need to cross the AD and GFAP-sgp130Fc strains), which shall decrease significantly Aβ production [28,49,50,51]; (ii) genetic background of the used strains [52]; (iii) differences in the housing conditions or in the used methodology [53]. Glial cells and neuroinflammation may have a critical role in the prevention, but also initiation and progression of AD [54] interfering in multiple brain functions as energy metabolism [3]. They are involved in the maintenance of the CNS homeostasis and protection from various stresses (i.e., hypoxia) by providing metabolic and structural support [55]. Regarding the role of IL-6 trans-signaling, previous studies demonstrated decreased neuroinflammation, including IL-6 production, in response to peripheral LPS if sgp130 is administered icv [56]; and using the same GFAP-sgp130Fc, a prominent down-regulation of the neuroinflammation caused by the transgenic expression of IL-6 was observed [24]. In agreement with that view, in the hypothalamus of 3xTg-AD male mice *Il6* gene expression was increased significantly, and this was blocked by sgp130Fc. Interestingly, blocking IL-6 trans-signaling increased the Aβ_42_/Aβ_40_ ratio in the hippocampus of female mice following HFD. 

## 5. Conclusions

The aforementioned evidences suggested a hypermetabolic state in the 3xTg-AD animals which is modulated, in a sex-dependent manner, by the inhibition of IL-6 trans-signaling. The reasons for these sex-specific differences are likely to be attributed to hormonal factors [57,58].
